# Adiponectin Inhibits LPS-Induced HMGB1 Release through an AMP Kinase and Heme Oxygenase-1-Dependent Pathway in RAW 264 Macrophage Cells

**DOI:** 10.1155/2016/5701959

**Published:** 2016-05-30

**Authors:** Mohamed Elfeky, Ryuji Kaede, Yuko Okamatsu-Ogura, Kazuhiro Kimura

**Affiliations:** ^1^Department of Biomedical Sciences, Graduate School of Veterinary Medicine, Hokkaido University, Kita 18, Nishi 9, Kita-ku, Sapporo 060-0818, Japan; ^2^Department of Biochemistry, Faculty of Veterinary Medicine, Alexandria University, Edfina, Behera 22785, Egypt

## Abstract

High mobility group protein B1 (HMGB1) is a late inflammatory mediator that exaggerates septic symptoms. Adiponectin, an adipokine, has potent anti-inflammatory properties. However, possible effects of adiponectin on lipopolysaccharide- (LPS-) induced HMGB1 release are unknown. The aim of this study was to investigate effects of full length adiponectin on HMGB1 release in LPS-stimulated RAW 264 macrophage cells. Treatment of the cells with LPS alone significantly induced HMGB1 release associated with HMGB1 translocation from the nucleus to the cytosol. However, prior treatment with adiponectin suppressed LPS-induced HMGB1 release and translocation. The anti-inflammatory cytokine interleukin- (IL-) 10 similarly suppressed LPS-induced HMGB1 release. Adiponectin treatment decreased toll-like receptor 4 (TLR4) mRNA expression and increased heme oxygenase- (HO-) 1 mRNA expression without inducing IL-10 mRNA, while IL-10 treatment decreased TLR2 and HMGB1 mRNA expression and increased the expression of IL-10 and HO-1 mRNA. Treatment with the HO-1 inhibitor ZnPP completely prevented the suppression of HMGB1 release by adiponectin but only partially inhibited that induced by IL-10. Treatment with compound C, an AMP kinase (AMPK) inhibitor, abolished the increase in HO-1 expression and the suppression of HMGB1 release mediated by adiponectin. In conclusion, our results indicate that adiponectin suppresses HMGB1 release by LPS through an AMPK-mediated and HO-1-dependent IL-10-independent pathway.

## 1. Introduction

Sepsis, an almost universally fatal clinical syndrome that is caused by microbial infection, results from excess stimulation of the host immune system by pathogen components to produce various proinflammatory cytokines [1]. Overproduction of these cytokines causes systemic inflammation that can lead to tissue damage, multiple organ failure, and death [2, 3]. For example, bacterial lipopolysaccharides (LPS), a cell wall component of gram-negative bacteria, induces an acute inflammatory response initiated by its interaction with toll-like receptor 4 (TLR4) resulting in sequential release of “early” (e.g., tumor necrosis factor- (TNF-) *α*, interleukin- (IL-) 1, and IL-6) and “late” (e.g., high mobility group protein B1 (HMGB1)) proinflammatory cytokines [4–6]. However, therapies designed to block early released cytokines such as TNF-*α* or IL-1*β* have shown limited efficacy due to the early and transient kinetics of the production of these inflammatory cytokines [7, 8].

HMGB1 is a highly conserved, ubiquitous nonhistone nuclear protein that exhibits diverse functions according to its cellular location. Nuclear HMGB1 participates in DNA replication, recombination, transcription, and repair. In response to infection or injury, HMGB1 is actively secreted by innate immune cells and/or passively released by injured or damaged cells. Once released, HMGB1 binds with cell-surface receptors, such as the receptor for advanced glycation end products (RAGE) and/or TLRs including TLR2 and TLR4 and mediates various cellular responses, infiltration of innate immune cells, and subsequent release of various proinflammatory cytokines [9–12]. Administration of recombinant HMGB1 to mice is lethal, while administration of anti-HMGB1 antibodies or inhibitors provides protection against LPS-induced acute tissue damage and lethal endotoxaemia [4, 11, 13, 14]. Therefore, targeting HMGB1 release provides a wide window for clinical intervention against systemic inflammatory diseases.

Adiponectin, which is also known as adipocyte complement-related protein (Acrp30), is one of the most abundant ones of the bioactive molecules called adipokines that are secreted from adipose tissue [15]. Adiponectin plays an important role in various physiological processes including lipid metabolism, insulin sensitization, and anti-inflammatory responses [16–18]. Evidence indicates that adiponectin suppresses the “early” phase of macrophage inflammatory responses. For example, adiponectin reduces macrophage differentiation and migration [19] and promotes macrophage polarization toward an anti-inflammatory M2 phenotype both in vivo and in cultured macrophages [20, 21]. Adiponectin also inhibits the upregulation of the expression of adhesion molecules and the enhancement of phagocytic activity and cytokine production in LPS-stimulated macrophages [19, 22], whereas it increases the release of anti-inflammatory mediators such as IL-10 and IL-1 receptor antagonist from macrophages [23].

A number of animal studies show that adiponectin has a protective effect against the development of inflammation related disorders. For example, treatment with adiponectin improves atherosclerosis through inhibition of macrophage aggregation [19] and improves nonalcoholic steatohepatitis via inhibition of lipogenic factors and TNF-*α* [24]. Moreover, adiponectin protects from endotoxin-induced disorders of organs including the liver [25], the lung [26], and the heart [27], although its deficiency is associated with severe polymicrobial sepsis with high mortality [28]. However, there has been no published report regarding the effects of adiponectin on the regulation of endotoxin-mediated release of “late” proinflammatory mediators such as HMGB1. Therefore, in this study, we investigated the effect of adiponectin on LPS-induced HMGB1 release in murine RAW 264 macrophage cells.

## 2. Materials and Methods

### 2.1. Materials

Rabbit anti-HMGB1 antibody was purchased from Cell Signaling Technology (CST) (Beverly, MA, USA). Recombinant mouse full length adiponectin expressed in HEK293 cells was purchased from Biovendor (Asheville, NC, USA). Recombinant murine IL-10 was purchased from PeproTech (Rocky Hill, NJ, USA). Zinc protoporphyrin IX (ZnPP) was purchased from Frontier Scientific (Logan, UT, USA). SB203580, compound C (dorsomorphin), wortmannin, and bovine serum albumin (BSA) were purchased from Sigma-Aldrich Fine Chemicals (St. Louis, MO, USA). OPTI-MEM I was purchased from Invitrogen (Carlsbad, CA, USA).

### 2.2. Cell Culture

Cells of the murine macrophage-like cell line RAW 264 (RCB0535, RIKEN Cell Bank, Japan) were maintained in Dulbecco's modified Eagle's medium (DMEM, Wako Pure Chemicals, Osaka, Japan) and supplemented with 10% fetal bovine serum (FBS, Trace Scientific Ltd., Melbourne, Australia), 100 U/mL penicillin, and 100 *μ*g/mL streptomycin in an atmosphere of humidified 5% CO_2_ at 37°C. When the cells reached 80–90% confluence, they were washed twice with, and subsequently cultured in, serum-free OPTI-MEM I for 12 h before all treatments. The cells were treated with or without increasing concentrations of adiponectin or IL-10 for 18 h and were then stimulated with LPS (*Escherichia coli* O55:B5, Sigma-Aldrich) dissolved in phosphate buffer saline (PBS) at a concentration of 200 ng/mL for another 24 h. When included, the cells were treated with or without ZnPP, SB203580, compound C, or wortmannin 1 h before adiponectin (10 *μ*g/mL) or IL-10 (100 ng/mL) addition.

### 2.3. Western Blotting Analysis

The level of HMGB1 in the culture medium was determined by western blotting analysis as previously reported [29–32]. Briefly, culture medium samples were centrifuged to remove cellular debris, then concentrated 60-fold with the Amicon Ultra-4-10000 NMWL (Millipore, Billerica, MA, USA). The concentrated samples were mixed with SDS loading buffer (500 mM Tris-HCl, 10% SDS, 0.5% bromophenol blue, and 5% 2-mercaptoethanol), boiled at 100°C for 5 min, separated on 15% SDS-polyacrylamide gels, and transferred onto a polyvinylidene fluoride membrane (Immobilon, Millipore). The membrane was incubated in a blocking buffer (20 mM Tris-HCl (pH 7.5), 150 mM NaCl, 0.1% Tween 20 (TBS-T), and 5% skimmed milk) and then with rabbit anti-HMGB1 polyclonal antibody (1 : 2000 dilution in the blocking buffer) overnight at 4°C. Subsequently, the membrane was washed with TBS-T for 15 min and incubated with horseradish peroxidase-linked goat anti-rabbit immunoglobulin (CST) (1 : 5000 dilution in the blocking buffer) for 1 h at room temperature. The signals were visualized using chemiluminescent HRP Substrate (Millipore) according to the manufacturer's instructions and were detected using the ImageQuant LAS 500 system (GE Healthcare, Buckinghamshire, UK). The intensity of chemiluminescence of the corresponding bands was quantified using Image J software (v. 1.48, http://imagej.nih.gov/ij/).

### 2.4. Quantitative Real-Time PCR (qRT-PCR)

Total RNA was extracted from RAW 264 cells using the RNAiso reagent (Takara Bio, Shiga, Japan) according to the manufacturer's protocol. Total RNA (2 *μ*g) was reverse transcribed using a 15-mer oligo (dT) adaptor primer and M-MLV reverse transcriptase (Invitrogen). Quantitative real-time PCR was performed on a fluorescence thermal cycler (Light Cycler system, Roche Diagnostics, Mannheim, Germany) using FastStart Essential DNA Green Master PCR kits (Roche Diagnostics). Expression levels were determined using the standard curve method with respective cDNA fragments as standards. The levels are reported relative to Gapdh expression as an internal control. The primer sequences used in this study and the length of each PCR product are listed in Table 1.

### 2.5. Immunofluorescence

The cellular localization of HMGB1 was investigated using an immunofluorescence staining assay. RAW 264 cells (5 × 10^4^ cells/well) were cultured on glass coverslips in 6-well plates. The cells were washed twice with PBS and then fixed with 4% paraformaldehyde for 30 min at room temperature. Subsequently, the cells were permeabilized with 10% Triton X-100 in PBS supplemented with 0.5% BSA and 0.15% glycine for 10 min, following which they were blocked in PBS containing 5% BSA and 0.3% Triton X-100 for 60 min. The glass coverslips were then incubated with rabbit anti-HMGB1 antibody (1 : 100 dilution in PBS containing 1% BSA and 0.3% Triton X-100) overnight at 4°C, followed by goat anti-rabbit Alexa flour 488 (1 : 400 dilution) (Invitrogen) in the dark for 1 h at room temperature. Cells were washed with PBS containing 0.1% Triton X-100 between all incubations steps, followed by a final wash in PBS. Nuclei were labeled by incubation with 4′,6-diamidino-2-phenylindole (DAPI, Invitrogen) for 10 min. The cells were washed three times for 5 min with PBS. The coverslips were mounted on slides using Prolong® Antifade Reagents (Invitrogen). Images were captured using a fluorescence microscope (Biorevo BZ-9000, Keyence Japan, Osaka, Japan) with a ×100 oil-immersion lens. No fluorescence was detected in control cells processed without the primary antibody. The fluorescence intensities of cytosolic and nuclear HMGB1 were quantified using Image J software.

### 2.6. Statistical Analysis

IBM SPSS Statistics version 22.0 software (SPSS, Chicago, IL, USA) was used for statistical analysis. Data are presented as means ± standard error (SE). Statistical comparisons between multiple groups were performed with one-way analysis of variance (ANOVA) followed by either Dunnett's or a Tukey HSD post hoc test. A *p* value of < 0.05 was considered statistically significant.

## 3. Results

**Figure 1:**
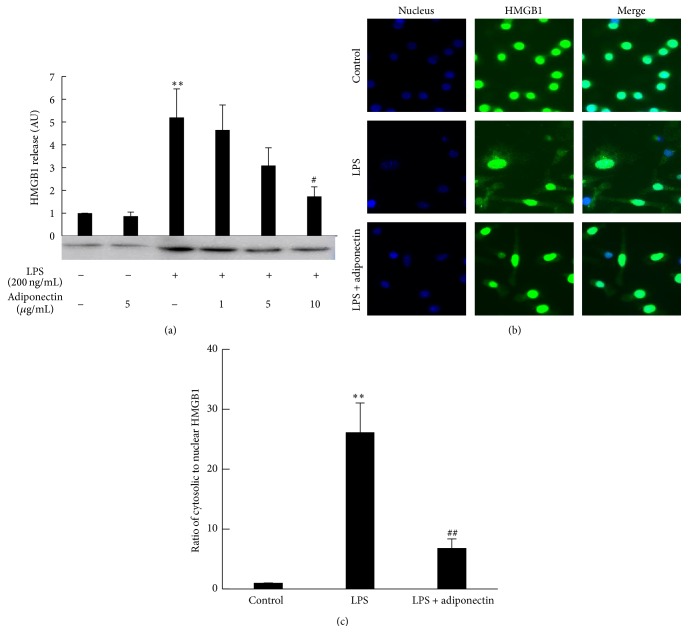
Effect of recombinant adiponectin on LPS-induced HMGB1 release and HMGB1 cellular translocation. Raw 264 cells were cultured in DMEM supplemented with 10% FBS and were cultured in serum-free OPTI-MEM I medium for additional 12 h. The cells were treated with increasing concentrations of adiponectin for 18 h, then stimulated with LPS (200 ng/mL) for another 24 h. (a) Culture medium was collected and analyzed by HMGB1 western blotting, followed by quantification of the intensity of the chemiluminescent HMGB1 band. The results are expressed as means ± SE of three independent experiments (^*∗∗*^ *p* < 0.01 significance compared with control; ^#^ *p* < 0.05 significance compared with LPS treated cells). (b and c) Cellular HMGB1 was immunostained with an anti-HMGB1 rabbit primary and Alexa Fluor 488 anti-rabbit secondary antibodies. The nucleus was stained with DAPI. Merge indicates the combination of both HMGB1 (Green) and nuclear (Blue) fluorescence. The fluorescence intensities of cytosolic and nuclear HMGB1 in (b) were separately analyzed and the ratio of cytosolic HMGB1 to nuclear HMGB1 is shown in (c) (^*∗∗*^ *p* < 0.01 significance compared with control; ^##^ *p* < 0.01 significance compared with LPS treated cells).

RAW 264 cells released a small amount of HMGB1 into the medium under the culture conditions without any stimulation. The amount of HMGB1 that was released increased 6-fold upon stimulation of the cells with 200 ng/mL of LPS (Figure 1(a)). Cell viability was almost 100% even after treatment with 1 *μ*g/mL of LPS (data not shown). The increased release of HMGB1 with LPS treatment was accompanied by HMGB1 translocation from the nucleus to the cytosol (Figures 1(b) and 1(c)). These results suggested that HMGB1 release was under the control of LPS signaling rather than being passive release due to LPS cytotoxicity. Prior treatment of the cells with full length adiponectin failed to affect basal HMGB1 release but dose dependently suppressed LPS-induced HMGB1 release and was accompanied by nuclear localization of most of the HMGB1 (Figures 1(a)–1(c)).

**Figure 2:**
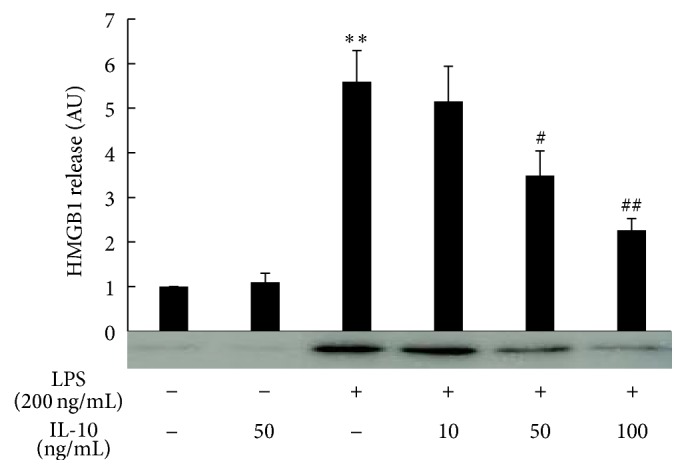
Effect of recombinant IL-10 on LPS-induced HMGB1 release. Raw 264 cells were cultured as described in Figure 1 legend and were treated with IL-10 for 18 h, then stimulated with LPS (200 ng/mL) for another 24 h. Culture medium was collected and analyzed by HMGB1 western blotting followed by quantification of the intensity of the chemiluminescent HMGB1 band. The results are expressed as means ± SE of three independent experiments (^*∗∗*^ *p* < 0.01 significance compared with control; ^##^ *p* < 0.01 and ^#^ *p* < 0.05 significance compared with LPS treated cells).

As it has been reported that globular adiponectin exerts its anti-inflammatory actions through induction of IL-10 [21], we next examined the effect of IL-10 on LPS induction of HMGB1 release. Prior treatment of the cells with IL-10 also failed to enhance basal HMGB1 release. However, IL-10 at doses of 50 and 100 ng/mL greatly decreased the HMGB1 release into the medium that was induced by LPS (Figure 2).

**Figure 3:**
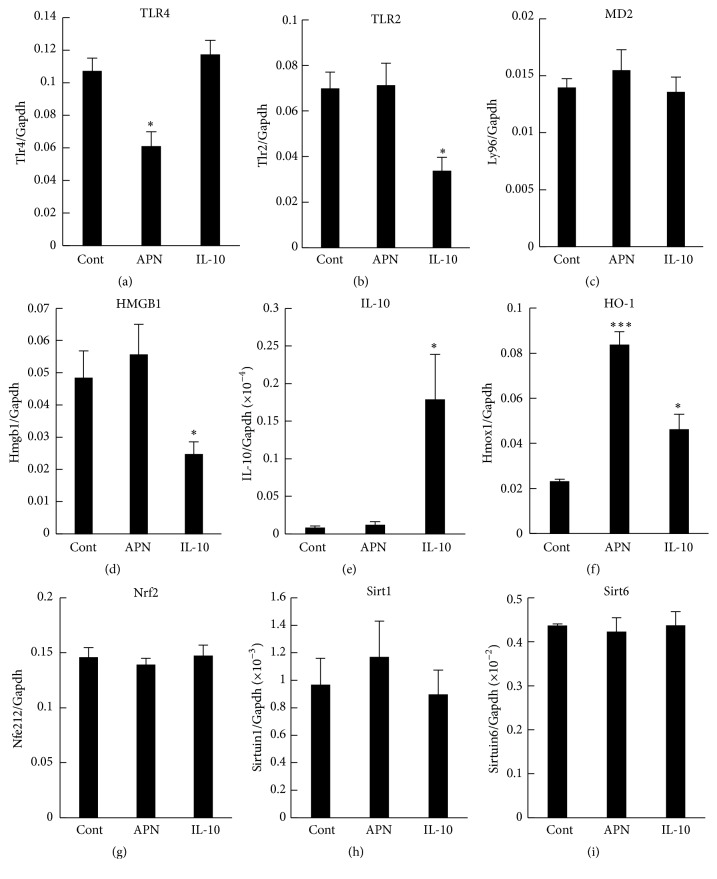
Effects of adiponectin and IL-10 on mRNA expression in RAW 264 cells. Raw 264 cells were cultured as described in Figure 1 legend and were treated with adiponectin (APN, 10 *μ*g/mL) and IL-10 (100 ng/mL) for 18 h. RNA was extracted, and expressions of (a) TLR4, (b) TLR2, (c) MD2, (d) HMGB1, (e) IL-10, (f) HO-1, (g) Nrf2, (h) Sirt1, (i) Sirt6, and Gapdh (control) mRNAs were measured using qRT-PCR. The results are expressed as means ± SE of three independent experiments (^*∗∗∗*^ *p* < 0.001 and ^*∗*^ *p* < 0.05 significance compared with control (Cont)).

To further examine the mechanism behind the suppressive effect of adiponectin on LPS-induced HMGB1 release, we compared mRNA expression in cells treated with either full length adiponectin or IL-10. Among the genes quantified, RAW 264 cells constitutively expressed TLR4 mRNA, TLR2 mRNA, and myeloid differentiation factor 2 (MD2) mRNA (Figures 3(a)–3(c)), all of which are plasma membrane components responsible for LPS binding and signaling. Treatment of the cells with adiponectin selectively decreased expression of TLR4 mRNA, while IL-10 treatment reduced only TLR2 mRNA expression. Distinct differences between full length adiponectin and IL-10 treatments were also observed in the expression of HMGB1 and IL-10 genes. The cells constitutively expressed HMGB1 mRNA, which was suppressed only by IL-10 treatment and not by adiponectin treatment (Figure 3(d)). On the other hand, the cells expressed very low levels of IL-10 mRNA, which was enhanced only by IL-10 treatment, but not by full length adiponectin treatment (Figure 3(e)). Thus, it was unlikely that full length adiponectin exerted its suppressive effect on LPS-induced HMGB1 release through induction of IL-10.

**Figure 4:**
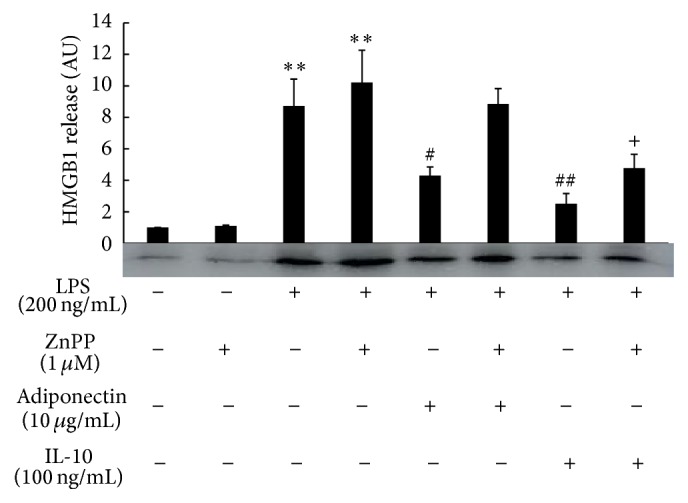
Effect of an HO-1 inhibitor on the anti-inflammatory action of adiponectin or IL-10 on LPS-induced HMGB1 release. Raw 264 cells were cultured as described in Figure 1 legend and were treated with dimethyl sulfoxide (DMSO, control) or ZnPP (1 *μ*M) for 1 h before treatment with adiponectin (10 *μ*g/mL) or IL-10 (100 ng/mL) for 18 h. Cells were then stimulated with LPS (200 ng/mL) for another 24 h. Culture medium was collected and analyzed by HMGB1 western blotting followed by quantification of the intensity of the chemiluminescent HMGB1 band. The results are expressed as means ± SE of three independent experiments (^*∗∗*^ *p* < 0.01 significance compared with control; ^##^ *p* < 0.01 and ^#^ *p* < 0.05 significance compared with LPS treated cells; ^+^ *p* < 0.05 significance compared with LPS plus ZnPP treated cells).

Interestingly, both IL-10 and full length adiponectin treatments enhanced the mRNA expression of HO-1, a downstream anti-inflammatory effector of IL-10 signaling (Figure 3(f)), while neither treatment affected the mRNA expression of nuclear factor erythroid-derived 2 related factor 2 (Nrf2), a transcription factor related with HO-1 gene expression (Figure 3(g)). In addition, neither IL-10 nor full length adiponectin treatment increased the mRNA expression of Sirt1 or Sirt6, which are histone deacetylases that function as a chromatin silencer to regulate recombination and genomic stability (Figures 3(h) and 3(i)). We therefore next examined the involvement of HO-1 in the suppressive effect of full length adiponectin on LPS-induced HMGB1 release. Treatment of the cells with zinc protoporphyrin (ZnPP), a HO-1 inhibitor, did not have any effect on HMGB1 release from either LPS-stimulated or control cells (Figure 4). However, treatment with ZnPP for 1 h before full length adiponectin treatment almost completely abolished adiponectin suppression of LPS-induced HMGB1 release, although it only slightly inhibited the suppression by IL-10. These results indicate that increased expression of HO-1 in response to full length adiponectin is necessary for adiponectin-mediated prevention of LPS-induced HMGB1 release.

**Figure 5:**
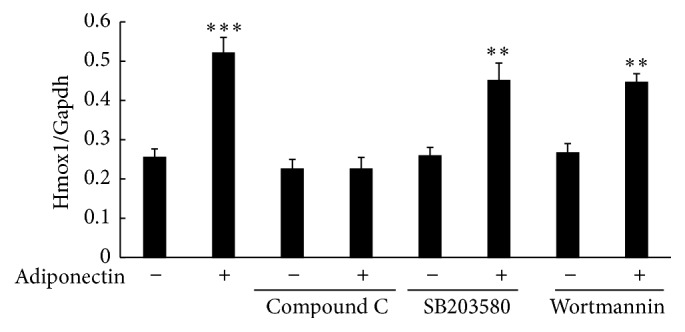
Effect of kinase inhibitors on adiponectin-induced HO-1 mRNA expression in RAW 264 cells. Raw 264 cells were cultured as described in Figure 1 legend and were treated with adiponectin (10 *μ*g/mL) for 18 h in the presence of DMSO (control), compound C (10 *μ*M), wortmannin (1 *μ*M), or SB203580 (10 *μ*M). RNA was extracted, and expressions of Hmox1 (HO-1) and Gapdh mRNAs were measured using qRT-PCR. The results are expressed as means ± SE of three independent experiments (^*∗∗∗*^ *p* < 0.001 and ^*∗∗*^ *p* < 0.01 significance compared with control).

**Figure 6:**
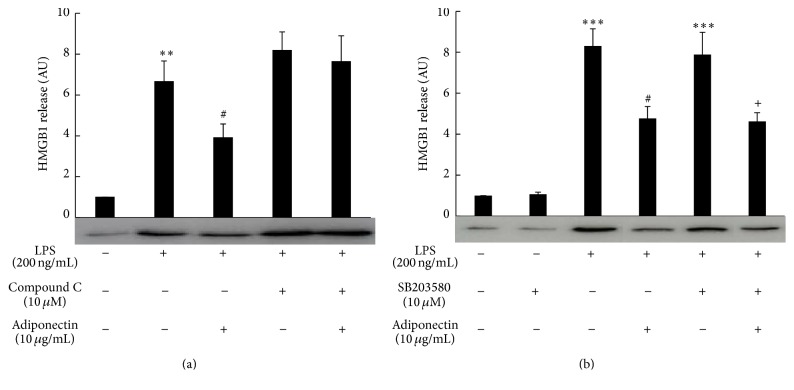
Effect of kinase inhibitors on the suppression by adiponectin of LPS-induced HMGB1 release. Raw 264 cells were cultured as described in Figure 1 legend and were treated with compound C (a) or SB203580 (b) for 1 h before treatment with adiponectin (10 *μ*g/mL) for 18 h, following which they were stimulated with LPS (200 ng/mL) for another 24 h. Culture media were collected and analyzed by HMGB1 western blotting followed by quantification of the intensity of the chemiluminescent HMGB1 band. The results are expressed as means ± SE of three independent experiments (^*∗∗∗*^ *p* < 0.001 and ^*∗∗*^ *p* < 0.01 significance compared with control; ^#^ *p* < 0.05 significance compared with LPS treated cells; ^+^ *p* < 0.05 significance compared with LPS plus SB203580 treated cells).

We then examined whether increased expression of HO-1 mRNA by full length adiponectin was mediated through AMP-activated kinase (AMPK), a main signaling pathway of adiponectin action [33–35]. Treatment of the cells with compound C, an AMPK inhibitor, abolished the increase in expression of HO-1 mRNA by full length adiponectin, whereas treatment of the cells with wortmannin, a phosphatidylinositol-3-kinase (PI3K) inhibitor, or with SB203580, a p38 mitogen-activated protein kinase (p38MAPK) inhibitor, did not affect this increase (Figure 5). Consistent with these results, treatment of the cells with compound C, but not with SB203580, abolished adiponectin-mediated suppression of LPS-induced HMGB1 release (Figure 6).

## 4. Discussion

In the present study, we demonstrated for the first time that full length adiponectin prevents LPS-induced HMGB1 translocation from the nucleus to the cytosol and its subsequent release from Raw 264 mouse macrophage cells. This process is most probably mediated by AMPK-dependent HO-1 induction, as evidenced by the following results. Both AMPK and HO-1 inhibitors prevented the suppression of LPS-induced HMGB1 release by full length adiponectin and the AMPK inhibitor also prevented induction of HO-1 mRNA by full length adiponectin. Furthermore, the mechanism of the full length adiponectin effect is supported by previous reports that showed that full length adiponectin activates AMPK activity [33–35], that activation of AMPK by metformin or dehydrodiconiferyl alcohol enhances HO-1 expression and its activity [36, 37], and that HO-1 is indispensable for the prevention of HMGB1 release [38, 39]. Of course, other events such as selective reduction in TLR4 mRNA expression by full length adiponectin might contribute, at least in part, to the suppression of LPS-induced HMGB1 release, since TLR4 is the predominant receptor for LPS [40, 41] and a similar decrease in cell-surface TLR4 expression is seen in macrophage cells treated with globular adiponectin [42]. However, other intracellular signaling pathways related to p38MAPK, PI3K, and the nuclear histone deacetylase sirtuin are unlikely to be involved in the mechanism, although they have been reported to be involved in some adiponectin functions [43–46] or in the processes of HO-1 induction and LPS-induced HMGB1 release [47–49].

We have also demonstrated that IL-10 is a potent inhibitor of LPS-induced HMGB1 release. However, the fact that full length adiponectin failed to induce IL-10 mRNA suggested that the suppression by full length adiponectin might not be attributed to IL-10 production. This hypothesis is supported by previous findings that the effects of full length adiponectin on macrophage function are independent of IL-10 [42, 44], although anti-inflammatory effects of globular adiponectin are mediated by IL-10 [21, 23, 50–52]. The discrepancy between the role of IL-10 in the effects of full length and globular adiponectin has not been explored but is possibly due to different signals mediated through adipoR2 and adipoR1, respectively [42].

Accumulating evidence indicates that HO-1 plays a pivotal role in the anti-inflammatory cytoprotective effects of a wide variety of compounds including statins, phytochemicals such as resveratrol, and aspirin [53]. HO-1 is a microsomal enzyme that catalyzes the degradation of proinflammatory free heme and produces equimolar amounts of carbon monoxide, bilirubin, and iron [54]. The mechanisms that mediate the anti-inflammatory effects of HO-1 are not fully understood, but the potent antioxidant activity of bilirubin and the signaling gas activity of carbon monoxide are reported to suppress apoptosis, necrosis, inflammation, and oxidative stress. Interestingly, HO-1 is induced by pathophysiological stimuli including LPS and hemodynamic changes, but in most cases pathophysiological activation of HO-1 results in only a transient or marginal increase in HO-1 that falls below the threshold necessary to activate downstream components such as carbon monoxide [53]. In the present study, a fourfold increase in HO-1 mRNA expression compared to its basal expression was induced by full length adiponectin, whereas only a twofold increase was induced by IL-10. Combined with the result that the HO-1 inhibitor only partially abrogated the suppression by IL-10 of LPS-induced HMGB1 release, these findings suggested that IL-10 induces only a marginal increase in HO-1 mRNA and mainly utilizes an HO-1-independent pathway for the suppression of HMGB1 release.

In summary, we provide the novel finding that full length adiponectin suppresses HMGB1 release by LPS through an AMPK-HO-1-dependent pathway. Therefore, adiponectin plays an important role as a regulator of inflammation through inhibition of both early and late proinflammatory mediators under pathological conditions such as sepsis. Thus, it is possible that adiponectin might be a target for development of therapeutic agents against sepsis and other systemic inflammatory disorders.

## Figures and Tables

**Table 1:** Primer sequences for quantitative real-time PCR and the length of each PCR product.

Mouse gene	Gene product	Foreword primer	Reverse primer	Product size (bp)
*Gapdh*	GAPDH	GAAGGTCGGTGTGAACGGATT	GAAGACACCAGTAGACTCCAC	294
*Hmgb1*	HMGB1	GGGAGACCAAAAAGAAGTTC	GGCAGCTTTCTTCTCATAGG	200
*Hmox1*	HO-1	TTCAGAAGGGTCAGGTGTCC	CAGTGAGGCCCATACCAGAA	193
*Il-10*	IL-10	GCCAAGCCTTATCGGAAATG	TTTTCACAGGGGAGAAATCG	163
*Ly96*	MD2	ACGCTGCTTTCTCCCATATT	CATTGGTTCCCCTCAGTCTT	150
*Nfe2l2*	Nrf2	ACATGGAGCAAGTTTGGCAG	TGGAGAGGATGCTGCTGAAA	235
*Sirtuin1*	SIRT1	AGGGAACCTTTGCCTCATCT	GAGGTGTTGGTGGCAACTCT	159
*Sirtuin6*	SIRT6	ACCTGCAACCCACAAAACAT	GGCTCAGCCTTGAGTGCTAC	178
*Tlr2*	TLR2	CGGAGGTAGAGTTCGACGAC	AACTGGGGGATATGCAACCT	127
*Tlr4*	TLR4	CAGCAAAGTCCCTGATGACA	AGAGGTGGTGTAAGCCATGC	179
